# Assessment of Age at the Stages of the Eruption of Third Molar Teeth among the People of North-Eastern India

**DOI:** 10.1155/2021/9714121

**Published:** 2021-12-30

**Authors:** Mahanta Putul, Ranjumoni Konwar, Malamoni Dutta, Bharati Basumatary, Madhab Chandra Rajbongshi, Kahua Das Thakuria, Bitumoni Sarma

**Affiliations:** ^1^Forensic Medicine and Toxicology, Assam Medical College and Hospital, Dibrugarh, 786002 Assam, India; ^2^Anatomy, Assam Medical College and Hospital, Dibrugarh, 786002 Assam, India; ^3^Radiology, Fakhruddin Ali Ahmed Medical College and Hospital, Barpeta, Assam, India; ^4^General Surgery, Tezpur Medical College and Hospital, Tezpur, 784153 Assam, India; ^5^Physiology, Tezpur Medical College and Hospital, Tezpur, Assam, India; ^6^School of Nursing, Mahendra Mohan Choudhury Hospital (MMCH), Guwahati, 781001 Assam, India

## Abstract

**Methods:**

A cross-sectional descriptive and analytical study was undertaken with 1060 Assamese individuals (642 males and 418 females) aged 14–26 years and was subjected to a clinical, dental, and general physical examination from January 2014 to December 2018. The data were statistically analyzed using Microsoft Excel and the Statistical Package for the Social Sciences (SPSS) version 22. The significant differences among variables were tested using the chi-square test and Student's *t*-test, considering a *p* value < 0.05 as significant.

**Results:**

The carried-out research showed no eruption (NE) status of M3 with an overall mean (±SD) age at 17.39 (±2.273) years, although a significantly lower age among males with a mean age of 16.92 (±2.138) years (*p* value < 0.001) was observed. The mean age (overall) for the complete eruption (CE) was observed at 20.33 (±2.566) years, which was seen earlier in males. The mandibular M3 appears earlier compared to the maxillary M3. The third molar eruption (TME) on both left and right quadrants of the jaw was observed substantially earlier in the lower jaw, compared to the upper jaw (*p* value < 0.025). The earliest CE of M3 was marked at 15 years. The differences in the frequencies of TME in different chronological age groups were found significant (*p* value < 0.001). A significant association between gender and TME (*p* value < 0.045) in the current study is worth noting.

**Conclusion:**

Thus, determined by TME as a valid method, age can be used for various purposes to establish a person's identity. Dental age estimated using third molar eruption status has a weighty association with chronological age. Thus, it should be utilized to determine the likely age of an individual.

## 1. Introduction

Due to the vast individual variation, determining a person's age for identification is often challenging for medical professionals. It is a crucial activity frequently required to be carried out by forensic medicine experts and other medical officers in general.

Age diagnosis is an overbearing job in recent times for various grounds, including disagreement with age certificates, and authentication of the same for different purposes in legal and civil matters [1] is required, including cases of illegal unaccompanied juveniles [2].

The chronological age (CA) follows a continuous and unstoppable course and is recorded as per the date of birth (DOB). It is to be established from the witness elements like parents, doctors, school teachers, and document details like birth certificate and passport [3]. Children of the same CA may show differences in the same biological systems. These have led to searching for a reliable biological age concept like the dental system's maturation stage [4] for age investigation.

Tooth developmental phases are significant biological markers for the CA approximation processes practised for investigative and anthropologic purposes [5–7]. Thus, tooth eruption is a parameter of developmental morphology that can be analyzed by clinical examination. The dental age (DA) is more reliable than the ages determined from bone development, and it shows less inconsistency than other developmental structures and little variability concerning CA [4].

In teenagers or individuals aged 20-30, third molars (M3) are the only developing teeth to be considered to resolve tooth age. The other approaches to assess age during this period are questionable [8]. Therefore, the development of M3 is helpful in age assessment for an individual of unknown CA [9], which can be used in forensic age investigations.

Furthermore, dental development is relatively independent of another maturation system through changes in dentition timing. The third molar eruption (TME) is one of the reliable indicators of age [10]. In addition, M3 development is the only available tool for estimating an individual's age after puberty in the whole process of dentition [11].

Since M3 has a very high interethnic variability, formulas calculated to estimate the age of a region from its developmental stages cannot be generalized to other populations [11]. It should be adjusted for each region. We, therefore, considered the current research to estimate the age of the eruption of M3 among the people of Assam.

## 2. Materials and Methods

Cross-sectional descriptive and analytical research was done in the Department of Forensic Medicine and Toxicology at Tezpur Medical College and Hospital, Assam, 2014-2018. The research participants (*n* = 1060) aged 14–26 years were selected randomly to evaluate the DA.

Healthy samples that were born that are the equal residence of Assam, since birth, without any history of surgery or trauma that is involved with the posterior quadrants of both jaws, impacted M3, nonsyndromic (Apert syndrome, ectodermal dysplasia, Hunter syndrome, etc.), and without any systemic disorder (hyperparathyroidism, celiac disease, musculoskeletal diseases, vitamin D resistant rickets, etc.) were only considered. Samples that are free from any known and visible congenital disorders, growth retardation, and without any diseases of teeth that may retard the tooth development were also included.

The participants' CA was obtained from their birth records, for instance, birth certificate, school identity card, driving license, and passport, with due verification and counselling. We did a general physical check-up to know any visible signs suggestive of congenital anomalies and pathological circumstances that hamper tissue maturation. The different TME phases were evaluated by clinical dental examination using standard dental instruments. If any part of the crown was evident through the oral mucosa, a tooth was considered as having erupted. As per the stages of the eruption of the third molar based on the clinical dental examinations, the patients were categorized as no eruption (NE) status if the third molar is not visible in any of the quadrants of the jaw, incomplete eruption (IE) status if any of the third molars have visibly erupted but not all four, and complete eruption (CE) status if all four third molars have erupted at the time of the study.

All the authors of the paper assessed the stages of TME by conventional clinical dental examination. The interobserver agreement was found to be 100% (Cohen's kappa) in a sample of 106 (10% of the total sample) selected individuals, which may be due to the apparent criteria for determining tooth eruption of the present study. Tooth calcification stages could not be assessed as the radiographic examination of the tooth was not undertaken in the present study.

The inclusion of 14-year-old participants in this study was to reconfirm the eruption time of the M3 among the people of this region.

Statistical analyses to evaluate any significant differences was done using the chi-square test and Student's *t*-test. Microsoft Excel (Microsoft Corporation, Redmond, WA) and the SPSS version 22 (IBM Corp., Armonk, New York) analyzed the data. A *p* value < 0.05 was considered significant. The ethics committee (human) of Tezpur Medical College and Hospital, Tezpur, Assam, India, has given the ethical approval vide ref: IHEC No 02/IEC/IMC/14 to conduct this study. Before data collection, informed consent was obtained from the participants.

## 3. Results

Out of 1060 total participants, 642 were male, and 418 were females. Among the participants, 60.7% were found to have no eruption (NE) of M3, while 23.1% had IE, and only 16.2% had CE with M3 in all quadrants.

The gender-wise analysis showed that the eruption of M3 was earlier in males compared to females as the mean age of NE status of the TME among males was substantially lower (16.92 ± 2.138 years) as compared to that of females with a mean age of 18.02 ± 2.303 years (*p* value < 0.001). No significant differences in the mean age of CE of M3 among males and females were observed, with an overall mean age of 20.33 years for clinically CE of M3 (Table 1).

Out of the 4240 M3, 1082 have been found visibly erupted during clinical examinations. Eruption of M3 was encountered more often in the mandible (620) than the maxilla (462). The current result shows the mean age of CE of maxillary M3 is 19.85 (±1.927) years in males and 19.83 (±2.918) years in female subjects, and that of M3 of the mandibular jaw is 18.52 (±2.371) years in male subjects and 19.31 (±1.811) years in female subjects. It is also observed that the mean age of IE of M3 was earlier in the right quadrant, specifically among males than females (Table 2).

The TME equally to the left and right quadrant of the jaws was observed significantly earlier in the lower jaw than the upper jaw (*p* value < 0.025), as shown in Table 3.

Table 4 displays the distribution of the stages of the TME on the CA. At the age of 14, all 38 (100.0%) participants showed NE. The TME occurred at an early age of 15 in 21 participants, among whom 3 (2.2%) participants showed CE. While 3 out of 11 participants belonged to the highest age of 26 years, none of the M3 erupted. The frequencies of TME in different CA groups were found statistically significant (*p* value < 0.001).

Table 5 demonstrates a statistically substantial relation between gender and TME (*p* value < 0.045). The CE and IE trend of M3 is more in male participants, whereas NE is observed more in females.

## 4. Discussion

Estimating age from TME is suggested because of the absence of other reliable biological markers during late adolescence [12], as all permanent teeth, except M3, have completed their formation by this time. Therefore, the eruption status of M3 was accessed to ascertain the participants' DA in the current study, as suggested by some other research studies [9, 12–14].

Earlier occurrence of complete TME in males compared to females was observed in the current study. Concurring the present result of a significant (*p* value < 0.05) earlier observation of the male's NE status of M3, compared to the female, another study [13] had reported the same findings.

Moreover, TME ages in the maxillary left were not significantly (*p* value > 0.536) different between males and females in the current study, supporting some works of the literature [15–17]. Just an early inclination of the eruption of M3 in the same quadrant was observed in females compared to the mean male age, supporting a scientific report [16]. The current results display the mean age of TME in the maxillary right quadrant as 17.71 (±1.939) years, consistent with some other works of the literature [15–17].

Besides, in line with a research report [16], females experiencing early TME with mean age 19.83 (±2.918) years compared to males mean age of 19.85 (±1.927) years was observed in the Maxillary Jaw (both upper).

In contrast, the TME in the mandibular jaw (both lower) occurred earlier in males with a mean age of 18.52 (±2.371) years compared to females with a mean age of 19.31 (±1.811) years. The overall mean age of TME was 18.81 (±2.207) years in the mandibular jaw, which is lower compared to TME in the maxillary jaw (both upper), at 19.84 (±2.302) years, agreeing to another study conducted among the South Indian population [13].

Furthermore, its earlier appearance in the female with a mean age of 17.71 (±1.113) years in the mandibular left quadrant compared to the mean male age of 18.54 (±2.340) years supports the results of another study [16].

The TME was observed earlier in females than the mean male age for both jaws' right halves, supported by some reports [15, 16]. These bilateral uneven and statistically insignificant (*p* value > 0.674) findings of TME concur with a research study [17].

Another research study [18] supports the overall mean age of 20.33 (±2.566) years of CE of M3 in all quadrants. Although variances of age among genders were not substantial (*p* value > 0.799), the prior observation in male participants than females agrees with some reports [15, 16].

The mean age of TME, either on the left or right side of both jaws, was observed earlier in the lower jaw. TME was substantially observed in the present study, earlier in the lower jaw than the upper jaw (*p* value < 0.025) in both the jaws' left and right sides. However, these findings contradict a recent research outcome [19].

In this study, the earliest complete TME was seen at 15 years, ranging from 15 to 26 years, which is in line with some reports [15, 16]. Contradicting these findings, studies reported the earliest TME at 17 years [20], with the eruption range as 18-24 years [18] and 18-21 years [21]. Some studies also advocated that their eruption time varies with race [21, 22]. However, the relation of DA estimated from the eruption status of M3 with CA in the present study was substantial (*p* value < 0.001).

The current study's higher frequency of CE of M3 observed in males than females agrees with an investigation [15]. The male preponderance in M3 eruption over the female confirms the trend reported by some reports [16, 23–25]. IE of M3 was also higher in males, whereas in the NE category, a higher frequency was observed in females. This variance was substantial. As reported in a review, these statistically significant (*p* value < 0.045) differences in frequency were influenced by sex [26, 27].

## 5. Limitation

The present study was carried out using only the clinical dental examination method. Radiological methods like X-rays and orthopantomograms were not used to assess the degree of calcification or the stage of development of M3. A broader study with advanced radiological techniques may help find the age at which the eruption of M3 starts occurring and the age at completion of the process, thus helping the investigators get a more accurate estimate of the age.

## 6. Conclusion

The eruption status of M3 provides valuable input in dental age estimation, which has a substantial relationship with the chronological age of an individual and can be used in age investigation.

Furthermore, it is established from the result that the eruption time of M3 varies from population to population. Hence, the TME time of one region should not be generalized to some other geographical areas.

## Figures and Tables

**Table 1:** Mean age at completion of the wisdom tooth eruption.

Status	Overall		Male		Female		*p* value
	No.	Mean ± SD	No.	Mean ± SD	No.	Mean ± SD	
No eruption (NE)	643	17.39 ± 2.273 (0.090)	371	16.92 ± 2.138 (0.111)	272	18.02 ± 2.303 (0.140)	<0.001^∗^
Incomplete eruption (IE)	245	18.67 ± 2.282 (0.146)	163	18.55 ± 2.430 (0.190)	82	18.90 ± 1.948 (0.215)	0.249
Complete eruption (CE)	172	20.33 ± 2.566 (0.196)	108	20.29 ± 2.547 (0.245)	64	20.39 ± 2.616 (0.327)	0.799

^∗^Statistically significant; figure in brackets is the standard error of the mean (SEM).

**Table 2:** Mean ages at third molar eruption in the quadrants of the jaw.

Status of eruption	Overall		Male		Female		*p* value
	No.	Mean ± SD	No.	Mean ± SD	No.	Mean ± SD	
Maxillary left	19	18.84 ± 2.141 (0.491)	12	19.08 ± 2.353 (0.679)	7	18.43 ± 1.813 (0.685)	0.536
Maxillary right	14	17.71 ± 1.939 (0.518)	9	17.33 ± 2.000 (0.667)	5	18.40 ± 1.817 (0.812)	0.344
Maxillary both	32	19.84 ± 2.302 (0.407)	20	19.85 ± 1.927 (0.431)	12	19.83 ± 2.918 (0.842)	0.985
Mandibular both	96	18.81 ± 2.207 (0.225)	61	18.52 ± 2.371 (0.304)	35	19.31 ± 1.811 (0.306)	0.092
Mandibular left	31	18.35 ± 2.138 (0.384)	24	18.54 ± 2.340 (0.478)	7	17.71 ± 1.113 (0.421)	0.377
Mandibular right	32	17.97 ± 2.456 (0.434)	21	17.76 ± 2.897 (0.632)	11	18.36 ± 1.286 (0.388)	0.519
Both right	21	18.19 ± 2.294 (0.501)	16	18.31 ± 2.549 (0.637)	5	17.80 ± 1.304 (0.583)	0.674

Statistically significant; figure in brackets is the standard error of the mean (SEM).

**Table 3:** Mean age at incomplete third molar eruption in the jaws.

	Upper jaw		Lower jaw		*p* value
	No.	Mean ± SD	No.	Mean ± SD	
Either left or right quadrant	33	18.36 ± 2.10 (0.336)	63	18.16 ± 2.29 (0.289)	0.670
Both left & right quadrant	32	19.84 ± 2.30 (0.407)	96	18.81 ± 2.21 (0.225)	0.025^∗^

^∗^Statistically significant; figure in brackets is the standard error of the mean (SEM).

**Table 4:** Dental age with chronological age.

Variables		Total	Complete eruption (CE)	Incomplete eruption (IE)	No eruption (NE)	*p* value
Chronological age in years	14	38	0 (0.0%)	0 (0.0%)	38 (100.0%)	<0.001^∗^
	15	139	3 (2.2%)	19 (13.7%)	117 (84.2%)	
	16	140	6 (4.3%)	24 (17.1%)	110 (78.6%)	
	17	190	21 (11.1%)	47 (24.7%)	122 (64.2%)	
	18	87	8 (9.2%)	29 (33.3%)	50 (57.5%)	
	19	129	25 (19.4%)	35 (27.1%)	69 (53.5%)	
	20	144	31 (21.5%)	40 (27.8%)	73 (50.7%)	
	21	100	32 (32.0%)	27 (27.0%)	41 (41.0%)	
	22	40	12 (30.0%)	13 (32.5%)	15 (37.5%)	
	23	20	14 (70.0%)	3 (15.0%)	3 (15.0%)	
	24	11	5 (45.5%)	6 (54.5%)	0 (0.0%)	
	25	11	9 (81.8%)	0 (0.0%)	2 (18.2%)	
	26	11	6 (54.5%)	2 (18.2%)	3 (27.3%)	

^∗^Statistically significant.

**Table 5:** Sexual dimorphism of the third molar eruption.

Variables		Total	Complete eruption (CE)	Incomplete eruption (IE)	No eruption (NE)	*p* value
Sex	Male	642	108 (16.8%)	163 (25.4%)	371 (57.8%)	0.045^∗^
	Female	418	64 (15.3%)	82 (19.6%)	272 (65.1%)	

^∗^Statistically significant.

## Data Availability

The data used to support the findings of this study are included in the article.

## References

[1] Macha M., Lamba B., Avula J. S. S., Muthineni S., Margana P. G. J. S., Chitoori P. (2017). Estimation of correlation between chronological age, skeletal age and dental age in children: a cross-sectional study. *Journal of Clinical and Diagnostic Research*.

[2] Pradella F., Pinchi V., Focardi M., Grifoni R., Palandri M., Norelli G.-A. (2017). The age estimation practice related to illegal unaccompanied minors immigration in Italy. *The Journal of Forensic Odonto-Stomatology*.

[3] Basaraba S. (2019). Defining chronological and biological age. *Healthy Aging*.

[4] Seth J., Agarwal A., Aeran H., Krishnan Y. (2018). Dental age estimation in children and adolescents. *Indian Journal of Dental Sciences*.

[5] Lewis J. M., Senn D. R. (2010). Dental age estimation utilizing third molar development: a review of principles, methods, and population studies used in the United States. *Forensic Science International*.

[6] Celik S., Zeren C., Çelikel A., Yengil E., Altan A. (2014). Applicability of the Demirjian method for dental assessment of southern Turkish children. *Journal of Forensic and Legal Medicine*.

[7] Stavrianos C., Mastagas D., Stavrianou I., Karaiskou O. (2008). Reviews. *Research Journal of Medical Sciences*.

[8] Bhat V. J., Kamath G. P. (2007). Age estimation from root development of mandibular third molars in comparison with skeletal age of wrist joint. *The American Journal of Forensic Medicine and Pathology*.

[9] Amin M. (2014). Western Saudi adolescent age estimation utilising third molar development. *European journal of dentistry*.

[10] Ogodescu E., Bratu E., Tudor A., Ogodescu A. (2011). Estimation of child's biological age based on tooth development. *Romanian Journal of Legal Medicine*.

[11] Khosronejad A., Navabi M. (2017). Correlation between chronological age and third molar developmental stages in an Iranian population (Demirjian method). *Dental Research Journal*.

[12] Monica T., Shraddha B., Balaji P. (2012). An evaluation of third molar eruption for assessment of chronologic age: a panoramic study. *Journal of Forensic Dental Sciences*.

[13] Priyadharshini K. I., Idiculla J. J., Sivapathasundaram B., Mohanbabu V. (2015). Age estimation using development of third molars in south Indian population: a radiological study. *Journal of International Society of Preventive and Community Dentistry*.

[14] Schmeling A., Olze A., Pynn B. R. (2010). Dental age estimation based on third molar eruption in First Nation people of Canada. *The Journal of Forensic Odonto-Stomatology*.

[15] Kuldeep S., Gorea R. K., Vipin B. (2005). Age estimation from eruption of permanent teeth. *Journal of Indian Academy of Forensic Medicine*.

[16] Acharya A. B., Ongole R., Praveen B. N. (2010). Clinical and radiological perspective of forensic dentistry. *Textbook of Oral Medicine, Oral Diagnosis and Oral Radiology*.

[17] Lewis A. J., Boaz K., Nagesh K. R. (2015). Demirjian's method in the estimation of age: a study on human third molars. *Journal of Forensic Dental Sciences*.

[18] Saini P., Pankaj J. P., Sharma V. K., Katara P. (2014). Prevalence of eruption status of third molars in college students of Bikaner (India). *International Journal of Medical Science and Education*.

[19] Deka S. J., Putul M., Nomi D., Neelutpal B., Jahnobi D., Thakuria K. D. (2019). Relationship between body mass index with eruption of third molar teeth. *International Journal of Health Research and Medico-Legal Practice*.

[20] Muller H. R. (1983). *Eine Studieüber die Inkonstanz des drittenMolaren (Fehlen, Anlage, Durchbruch)*.

[21] Elsey M. J., Rock W. P. (2000). Influence of orthodontic treatment on development of third molars. *The British journal of oral & maxillofacial surgery*.

[22] Kruger E., Thomson W. M., Konthasinghe P. (2001). Third molar outcomes from age 18 to 26: findings from a population-based New Zealand longitudinal study. *Oral Surgery, Oral Medicine, Oral Pathology, Oral Radiology, and Endodontology*.

[23] Olze A., Peschke C., Schulz R., Shmeling A. (2008). Studies of the chronological course of wisdom tooth eruption in a German population. *Journal of Forensic and Legal Medicine*.

[24] Olze A., Ishikawa T., Zhu B. L. (2008). Studies of the chronological course of wisdom tooth eruption in a Japanese population. *Forensic Science International*.

[25] Brkiæ H., Vodanoviæ M., Dumanciæ J., Lovriæ Z., Cukoviæ-Bagiæ I., Petrovecki M. (2011). Chronology of third molar eruption in Croatians. *Collegium Antropologicum*.

[26] Obuekwe O. N., Enabulele J. E. (2017). Gender variation in pattern of mandibular third molar impaction. *Journal of dental health, oral disorders & therapy*.

[27] Schmeling A., Grundmann C., Fuhrmann A. (2008). Criteria for age estimation in living individuals. *International Journal of Legal Medicine*.

